# Dental Policy Lab 2 - towards paying for health in dentistry

**DOI:** 10.1038/s41415-021-3725-1

**Published:** 2021-12-17

**Authors:** Marco E. Mazevet, Nigel B. Pitts, Catherine Mayne

**Affiliations:** Faculty of Dentistry, Oral & Craniofacial Sciences, King´s College London, Tower Wing, Guy´s Hospital, London, SE1 9RT, UK

## Abstract

The first Alliance for a Cavity-Free Future (ACFF)/King's College London Dental Policy Lab, held in 2017, identified the need for a review of dental payment systems in order to see progress towards achieving improvements in caries and cavities. The lack of incentivisation for preventive intervention and care has long been a barrier to progress. The second Dental Policy Lab, held in July 2018, focused on this issue with the overarching question: 'How can we create and implement acceptable prevention-based dental payment systems to achieve and maintain health outcomes?' Using a design approach and participatory research, 29 participants from five stakeholder categories developed a blueprint report that aims to serve as a framework to adapt or create remuneration systems that are compatible with evidence-based dentistry with a focus on preventive care. Aimed at policymakers and policy entrepreneurs, this blueprint provides guidance and potential solutions using several international examples. The report and accompanying infographic explored in this paper have been well received and have helped to frame discussions in several country settings, with a direct implementation which is being trialled in France in 2021.

## Key points

- Policy Labs methodology can be used to facilitate innovative policies for highly complex, systemic issues.
- There are many opportunities to create hybrid dental payment systems that can be adapted for specific objectives.
- The third ACFF/King's College London Dental Policy Lab identified key elements which must be considered in order to create acceptable and implementable dental payment systems for different stakeholders.

## Introduction

### Overview

Untreated caries is the most prevalent non-communicable disease (NCD) in the world.^1^ A major barrier to making progress towards reducing caries levels is the lack of suitable, preventively oriented dental payment systems. Widely, dental teams around the world continue to be remunerated for 'drilling and filling', not rewarded for the non-surgical and preventative care and advice that would keep patients cavity-free. The creation and implementation of new payment systems to support preventive, non-surgical and tooth-preserving care is a huge policy challenge which, if achieved, would be a huge step towards encouraging a shift towards a preventively oriented dental workforce.^2^

## Materials and methods

Following on from the successful 2017 Dental Policy Lab (DPL1) 'Towards a cavity-free future: how do we accelerate a policy shift towards increased resource allocation for caries prevention and control?'^3^ hosted by the Alliance for a Cavity-Free Future (ACFF) and King's College London, discussion began around developing on the four key outcomes from that event:

- Demonstrate the value of a cavity-free world to professionals, the public and policymakers
- Create prevention-based payment systems
- Better equip the dental and healthcare workforce
- Shift public and industry behaviours.

The overwhelming consensus from the DPL1 participants that the creation of effective remuneration systems which reward preventive intervention was a key step in realigning the focus of systems and practitioners led to the development of the concept for a second Dental Policy Lab (DPL2), held in July 2018, titled 'Towards paying for health in dentistry: how can we create and implement acceptable prevention-based dental payment systems to achieve and maintain health outcomes?' The methodology in the design of DPL2 was similar to that used for DPL1. The previously developed Win6 Cube^3^ was helpful as a structural tool, with the six stakeholder groups identified requiring consideration within all discussion on framing systems thinking, with the need to address the impact of reforms or system changes on each of the groups.

In addition to a core facilitation team, a Health Economics working group was included in the design process for DPL2, as the nature of the question being targeted demanded specialist input into the development of the activity and focus points for the Lab. The outcomes of this working group were highly motivating. Given the requirements of the Lab, both in terms of understanding of the field and commitment to working from participants, it was surmised that participatory research and design thinking would be a very appropriate tool to tackle this subject. The lists of questions developed by the Health Economics Working Group were integrated in the preparation of the briefing pack and ultimately aided in the formation of the exercises undertaken during DPL2.

This preliminary work synthesised the scientific evidence and broke down the discussion issues to effectively build the shape of the Lab and enable the included group work to be accessible by all parties. DPL2 involved the contributions of 29 participants who were selected using the Win6 Cube^3^ as a framework. These included representatives of practitioner and provider associations, government officials and chief dental officers, public health specialists, professional guidance specialists, payers (public and private) and the oral health industry. Given the nature of the subject, several health economists were also invited to participate and provide their expertise.

As well as presentations and lectures on relevant topics, iterative workshops and group work were undertaken by participants across the 24 hours, which had the objective of drawing out the key components needed in the creation of a blueprint for prevention-based payment systems. Relevant recent developments and trends were identified, along with previous barriers to progress and key insights to create a mind map framing the discussion topic by systems thinking.

### Stakeholder group development

In order to ensure that outcomes were relevant across the range of required stakeholders, all question topics were approached from the perspective of each of the stakeholders identified by the Win6 Cube, with a view to identifying further specific challenges and areas where rapid progress might be made towards the creation of a viable payment model, as well as key areas of focus.

The following is an example list of the 'government' stakeholder group priorities drawn out from one of the exercises.

From the perspective of governments, a prevention-based payment model should:

- Maintain access to the dental service
- Avoid perverse incentives (for example, cherry picking of high-risk patients)
- Enable recording of outcomes and costs
- Measure the impact on other diseases (NCDs) and possibly enable an interprofessional payment system
- Reduce inequalities
- Be realistic about the changes needed to invest in training and educating practitioners
- Be compatible with the workforce (now and projected)
- Be compatible with the Minamata phasedown of amalgam/phase up of prevention
- Be politically acceptable
- Achieve buy-in from professionals and avoid conflicts with trade unions.

## Results

Drawing together the thinking of all groups and discussion during this workshop, the following items were identified as key elements in successful payment system design:

- Identifying and measuring health outcomes
- Ensuring access and avoiding discrimination
- Understanding and shifting patient attitudes and behaviours
- Supporting practice-level sustainability
- Assuring system-level sustainability
- Incentivising quality and innovation
- Putting in place the data required
- Taking the lead as a profession.

Simple outlines were drawn up for each, addressing the more focused issues raised within each topic, with agreement on headline messaging and key issues to be addressed within the system design process. As a summary exercise, participants were given 'payment tokens', and were tasked with placing proportional value onto each of the elements and ideas presented.

### Report

Following the DPL2 meeting, the outcomes from the Policy Lab process and results from the valuation exercise were analysed and synthesised into a 20-page, multi-stakeholder-oriented report, published in 2019.^2^^,^ Similar to the DPL1 report,^3^ the second report is voluntarily short, graphical and aimed at policymakers for advocacy.

The key components outlined in the report are listed below.

#### What we should pay for

Standardised and measurable health outcomes; innovative and evidence-based preventive interventions; personalised and integrative care; paying dentists for preventive and non-surgical interventions.

#### Who the system must work for

For patients; for professionals and providers; for government and payers.

#### How we deliver the change needed

Taking the lead as a dental profession; working collaboratively using multi-stakeholder approaches; establishing consistent standards; essential data should be comparable internationally, but also allow variations according to local requirements; adapting the blueprint for different types of dental health systems around the world.

#### What should be done next

Continue to build the collaborative network driving this change; expand and share the evidence base; test the model in different systems; develop implementation blueprints for a 'glocal' approach (using global evidence, adapted for local implementation).

## Discussion

### Format of the Policy Lab

Although the timespan was short, the 24-hour format over two days was deemed appropriate due to the challenges which may be found in mobilising experts for any longer periods.

The planning process of DPL2 was developed slightly following DPL1 and included the utilisation of a preliminary Health Economics Working Group in the development of Lab content. The preliminary working group session helped frame the discussions and the methodology proposed, particularly the focus on separating participants into stakeholder groups. This preliminary work was an important exercise for all facilitators to understand the complexities of system changes.

### Representativeness of the participants

Following feedback relating to DPL1, during the planning of DPL2, careful attention was given to participants including representatives from a range of countries including the US, Japan, Brazil, Israel, Turkey and Singapore, with the aim of ensuring a broad range of experience was drawn upon. It has since been further noted that the countries represented at DPL2 could also be considered as mostly high-income ones, and further attention could have been given to lower-middle-income countries for representation within the process.

The inclusion of patient representation during the Policy Lab process was considered, but due to the international objectives for this blueprint creation, it was argued that patient contributions might be prone to reflecting country-specific issues. It was therefore decided not to include them at this upstream stage but noted that patients should be included in local design and implementation of the payment systems.

### Proximity to government

Although many participants were official representatives of their countries, with many Chief Dental Officers in attendance alongside members of other groups such as trade unions, no conflict of interest was witnessed during DPL2. The format allowed free speech within the group discussion, which may be attributed to the decision to create a common blueprint, rather than singling out any countries. This is a good demonstration of how Policy Labs facilitate communication between stakeholders who may be traditionally opposed or have potentially conflicting interests. Caution would be required if this exercise were to be repeated in a local country setting.

### Payment systems blueprint

The objective of DPL2 was to create, with a participatory approach, a blueprint for prevention-based payment systems. As demonstrated by the report, although different types of payment systems may be considered, which system(s) should be implemented must be decided based on local requirements. This might be influenced by numerous factors, such as the presence of third-party payers, existing payment devices, the workforce structure, the objectives to be reached, the information systems in place and additional parameters invoked in the preliminary stages of DPL2.^2^

**Fig 1:**
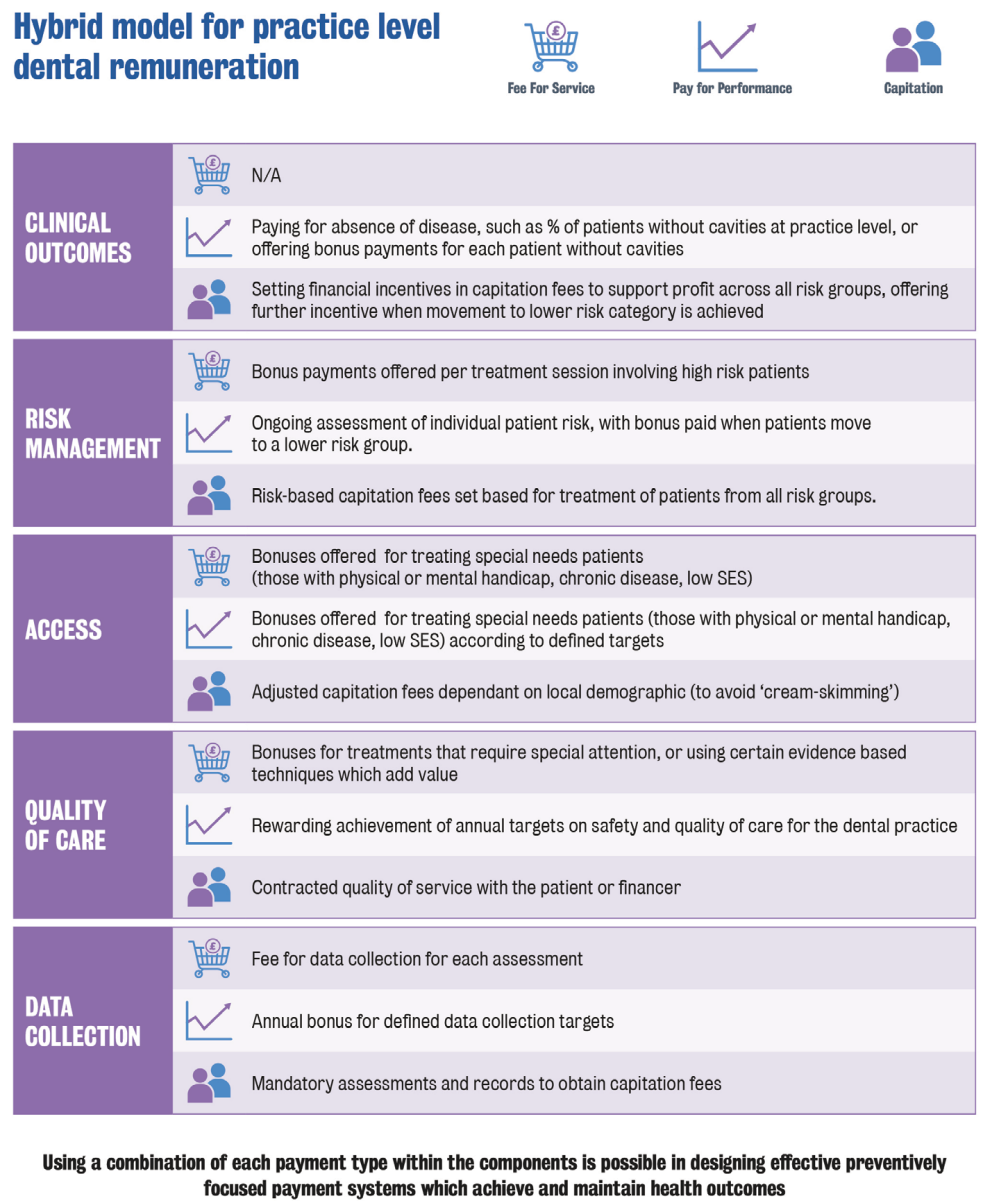
Possible combinations for hybrid payment systems, reproduced with permission from King's College London^2^

As our rapid review of the literature has shown, much attention has been given to defining and evaluating whether fee-for-service, capitation, pay-for-performance, salary or a combination of these could be considered more effective.^4^ However, such research is unlikely to show answers which can be extrapolated across different countries. As the DPL2 report indicates, there is a significant number of combinations that can be proposed, which must be analysed, if possible, through participatory research when designing a new system. Hybrid payment systems may have a better chance of achieving the desired outcomes, though robust evidence is still lacking both in the oral and general healthcare settings.^5^^,^^6^ A few examples can be found in Figure 1.

Some payment system characteristics can overlap, and based on the desired outcomes, one or more may achieve the same results with the help of mechanisms such as price adjustments. As an example, some at-risk populations in certain system types benefit from zero out-of-pocket charges on dental treatments as they are state-funded.

A high fee-for-service remuneration (high-incentive for providers) on treatments for these patients might be equally as effective as capitated fees at leading to improved access, by encouraging the providers to treat them. Similarly, high fee-for-service remuneration for preventive interventions, such as risk identification and the management of risk factors, could be as effective as setting specific targets in pay-for-performance systems. Economic considerations and a deep understanding of the current situation in which the payment system is to be designed and implemented seem necessary to analyse how the providers might react to changes.

Despite the need for further development, the initial DPL2 blueprint was robust enough to support direct impact including the 2019 redevelopment of payment models in Belgium for both dentists and dental hygienists to focus on remuneration for preventive care, and to provide guidance and advocacy tools to start an experimentation on 600 dental practices in France.

### The French Experimentation

Intensive work led by 'Les CDF' (the largest dental trade union in France) in the 18 months following DPL2 resulted in a National Experiment being agreed between the French Government, the national health insurance and the dental trade unions. This work has been agreed under Article 51 legislation (a national health innovation scheme) and will test use of an adaptation of the payment model for dental care developed by the DPL2, in combination with the CariesCare International caries management model.^7^ This project will involve 15,000 patients aged 18 to 21 years old with over 600 dentists.^8^ The national agreement includes a review after three years, with a view to adaption for attempting country-wide implementation. Practitioners will be asked to classify patients with a red/amber/green strategy according to their caries risk,^7^ a basic periodontal examination and a basic erosive wear examination.^9^

A lump sum will be paid annually to the providers based on these risk groups (€120 for the green, €200 for the amber and €275 for the red risk groups) in order to keep these patients from developing new oral diseases. This system will be added on top of the already existing fee-for-service payments that exists in France, which guarantees zero out-pocket charges (co-payments) on most dental procedures.^10^ Payments have been calibrated to ensure that there is no financial incentive for providers to act surgically when these treatments can and should be avoided. An economic evaluation will be performed by an external auditor, which will match each included patient with a similar individual in terms of dental treatments experience, socioeconomic status, age and location. A bespoke online information system that collects clinical data, patient-reported outcome measures and practitioner feedback has been developed for the purpose of this study.

### An important note on outcomes

Strong emphasis was given in the DPL2 report to measuring health outcomes, but as of its publication, there was no consensus regarding which outcomes should be measured, despite attempts, such as the OuTMaC protocol^11^ or Core Outcome Set Initiative.^12^ Much debate in the literature regarding clinical outcome measures, patient-reported outcome measures related to the quality of life, willingness to pay or utility measures still persists.

An FDI World Dental Federation consensus project has worked to generate agreement among experts on this topic. This led to the publication in July 2020 outlining the development of an adult oral health standard set (AOHSS) composed of 31 criteria/outcome.^13^ Based on the uptake of this set of outcomes, its clinical validation and its feasibility, a move towards standardised outcomes might help policymakers globally.

## Conclusion

The Policy Lab methodology has shown to be a powerful tool for participatory research at the policy level. A comprehensive report was published, aimed at policymakers and other professional leaders, to create or change payment systems. It provides a blueprint that integrates multi-stakeholder requirements and components which must be considered when designing or implementing new remuneration strategies. The blueprint was specifically designed to be adaptable to multiple country configurations, keeping in mind that standardised and comparable data collections of both health outcomes and costs were a critical element that could help future research agendas, as well as understanding the cost-effectiveness of these new payment systems. The inclusion of an overview infographic (Appendix 1) which summarises the Dental Policy Lab process and its key recommendations was well received. This report, infographic and blueprint have proven to be successful tools and DPL2 has already led to substantial impact, particularly in the economic arena, and aided in the creation internationally of systems to pay dental practitioners for prevention as well as surgical care, addressing a longstanding barrier to providing optimal caries care.

**Figure:**
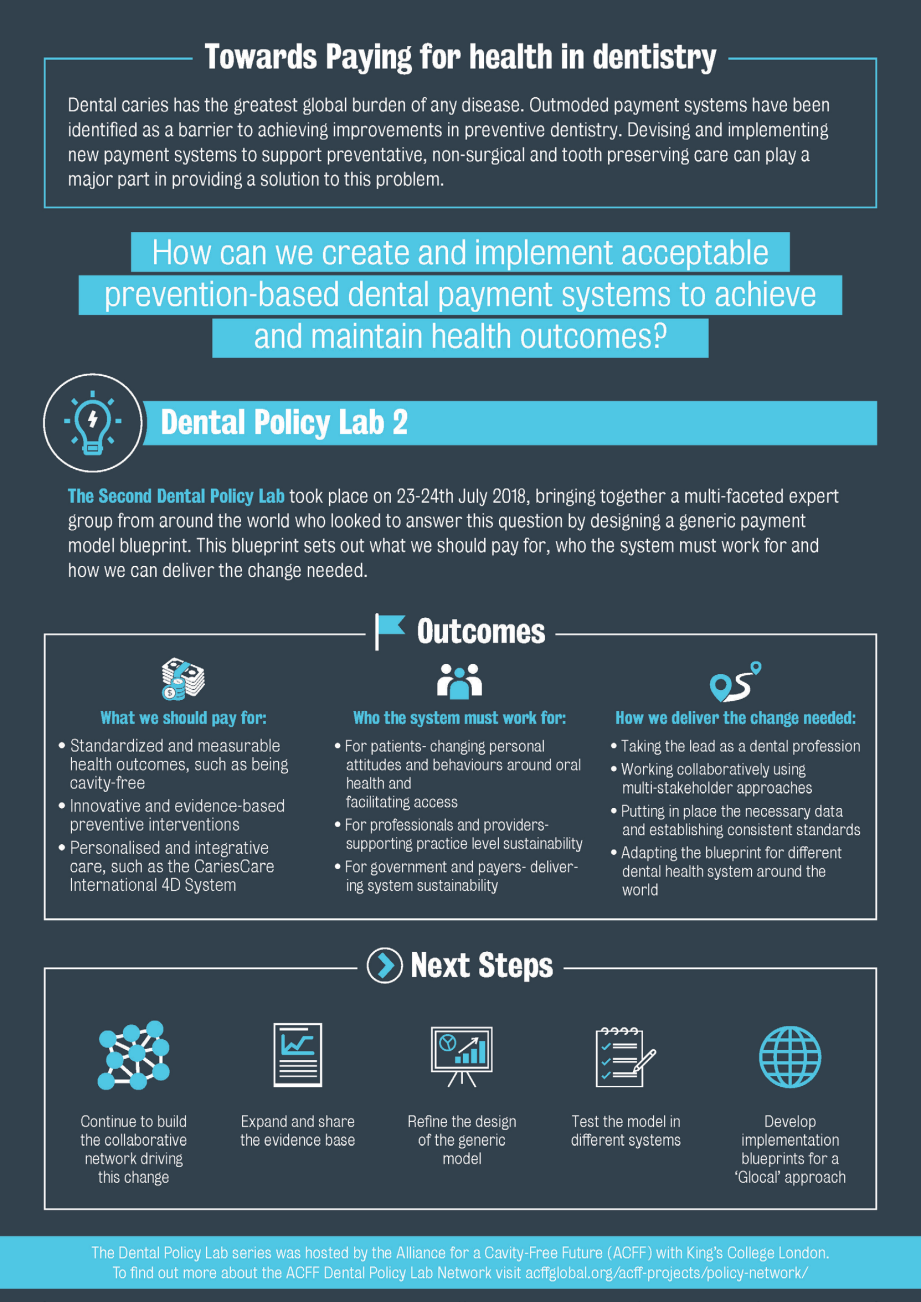
***Appendix 1*** Dental Policy Lab 2 overview infographic^2^
